# Recent Advances in Regenerative Endodontics: A Review of Current Techniques and Future Directions

**DOI:** 10.7759/cureus.74121

**Published:** 2024-11-20

**Authors:** Firas A Alothman, Lamia S Hakami, Ali Alnasser, Faris M AlGhamdi, Abdullah A Alamri, Basel M Almutairii

**Affiliations:** 1 Dentistry, Imam Abdulrahman Bin Faisal University, Dammam, SAU; 2 Dentistry, Private Clinic, Khobar, SAU; 3 Endodontics, Private Clinic, Khobar, SAU

**Keywords:** 3d bioprinting, biomaterials, clinical protocols, gene therapy, pulp-dentin complex, regenerative endodontics, scaffold development, signaling molecules, stem cells, tissue engineering

## Abstract

Regenerative endodontics is a rapidly evolving discipline focused on biologically restoring the pulp-dentin complex to revive vitality in non-vital teeth. Unlike traditional endodontic therapies that rely on inert materials to preserve structure, regenerative techniques aim to re-establish natural structure and function by harnessing advancements in tissue engineering. This narrative review examines recent progress in stem cell applications, scaffold development, signaling molecules, and clinical protocols that contribute to successful regenerative outcomes. Advances in stem cell sources, biomimetic scaffolds, and growth factor delivery systems have shown promising results, though challenges such as variability in outcomes and the need for standardized clinical protocols remain. This review also highlights future directions, including gene therapy and three-dimensional bioprinting, which hold the potential to overcome current limitations and pave the way for effective and reliable biologically restorative dental treatments.

## Introduction and background

Endodontics has traditionally focused on treating pulp disease and trauma by removing infection and preserving the remaining tooth structure. Root canal therapy and apexification, while effective at controlling infection and preserving teeth, result in the loss of the tooth’s natural biological functions, leaving it in a non-vital state. Teeth treated with these conventional approaches lack the sensory, immune, and regenerative functions inherent to vital pulp tissue. Over time, this loss of vitality can increase susceptibility to fractures and structural failure, as non-vital teeth often become brittle due to the lack of blood flow and cellular activity. The limitations of traditional treatments have highlighted the need for new therapeutic approaches that not only eliminate infection but also restore the tooth’s natural structure and function [1].

Regenerative endodontics has emerged as a groundbreaking field that shifts the focus from merely treating or preserving the tooth to actively regenerating the damaged or necrotic pulp tissue. Unlike traditional treatments that rely on inert materials to occupy the root canal space, regenerative endodontic procedures (REPs) aim to biologically restore the tooth’s pulp-dentin complex, allowing it to regain functions such as sensory responses, immune protection, and reparative capabilities. This regenerative approach is especially promising for cases involving immature permanent teeth with necrotic pulp, where conventional treatment options are limited and often result in compromised long-term outcomes. The concept of regenerating lost or damaged tissues within the tooth aligns with a broader movement in dentistry toward biological restoration, focusing on reestablishing natural structures and functions to improve oral health outcomes [2].

Regenerative endodontics is deeply rooted in the principles of tissue engineering, an interdisciplinary field that combines cell biology, biomaterials, and molecular biology to develop biological replacements for damaged tissues. The foundation of regenerative endodontics rests on the “tissue engineering triad,” which consists of three essential components: stem cells, scaffolds, and signaling molecules. Each element of this triad plays a unique role in promoting the formation of a living pulp-dentin complex. Stem cells serve as the cellular foundation for tissue formation, possessing the ability to differentiate into odontoblast-like cells essential for the regeneration of dentin and pulp tissues. Scaffolds provide the structural framework that supports cellular growth and organization while signaling molecules guide cell differentiation, tissue maturation, and vascularization. Together, these components aim to create a biological environment within the root canal that supports the regeneration of pulp tissue and enhances the tooth’s long-term resilience [3].

Historically, the idea of regenerating dental tissues has been explored for several decades, but it was only in the early 2000s that significant progress began to materialize. Initial research demonstrated that stem cells derived from dental pulp and apical papilla possess regenerative potential and are capable of differentiating into odontoblast-like cells under the right conditions. These discoveries have since spurred extensive research into the use of dental stem cells for pulp-dentin complex regeneration [4,5]. Multiple stem cell sources, such as dental pulp stem cells (DPSCs), stem cells from apical papilla (SCAPs), and dental follicle progenitor cells (DFPCs), have shown the capacity to generate key cellular components needed to reconstruct the pulp-dentin complex. These findings have opened new avenues for regenerative treatments that aim to reestablish a biologically active pulp tissue, which is integral to the tooth’s long-term health and functionality [6,7].

Regenerative endodontic therapy is currently applied primarily in cases of immature permanent teeth with necrotic pulp, where traditional treatments, such as apexification or pulpectomy, fail to promote continued root development and can lead to structurally weakened teeth. In contrast, regenerative endodontics can stimulate cellular activity that encourages root maturation and dentin formation, significantly enhancing the tooth’s structural integrity. In clinical trials and case studies, REPs have demonstrated promising results, showing improved outcomes in root development, apical closure, and thickness of dentin walls. These outcomes suggest that regenerative endodontics may offer a more viable, long-term solution for preserving immature teeth with necrotic pulp [8].

However, despite the promising advances, regenerative endodontics faces several significant challenges before it can be widely adopted in clinical practice. One of the primary issues is the lack of standardized treatment protocols. The regenerative outcomes of REPs are often variable and depend on numerous factors, including the choice of scaffold materials, the viability of stem cells, and the disinfection protocols used. Effective disinfection is crucial to prevent re-infection and ensure the success of the regenerative procedure, yet harsh disinfection agents can compromise the survival of stem cells and thus the overall regenerative outcome [9]. Additionally, ensuring predictable outcomes remains a significant hurdle, as the biological processes involved in pulp-dentin regeneration can be inconsistent and influenced by factors such as patient age, immune response, and individual biological variability [10].

Beyond procedural challenges, there is also the need for advancements in the materials and methods used in regenerative endodontics. Innovations in biomaterials and scaffold design are critical for creating supportive environments that promote cellular adhesion, differentiation, and tissue organization. Emerging technologies, such as 3D bioprinting, have the potential to produce highly customized scaffold structures that better mimic the natural extracellular matrix of dental pulp, enhancing cell alignment and differentiation [11]. Additionally, gene therapy and controlled-release growth factor systems represent promising areas of research that could improve the effectiveness and consistency of REPs by providing precise control over cellular behavior and tissue growth [12].

This review aims to provide a comprehensive overview of recent advances in regenerative endodontics, highlighting the theoretical underpinnings and practical applications of current techniques. It will examine the core components of the tissue engineering triad as they relate to endodontics, with an emphasis on innovations in stem cell therapies, scaffold materials, and signaling molecules. The review will also discuss clinical protocols and disinfection methods crucial to ensuring the success of REPs. By evaluating recent findings and identifying ongoing challenges, this review seeks to outline future directions in regenerative endodontics, including the potential roles of 3D bioprinting, gene therapy, and translational research. Ultimately, regenerative endodontics holds the promise of biologically restoring damaged or necrotic pulp tissue, offering a new path forward in the preservation and rehabilitation of non-vital teeth [13].

## Review

Methods

Literature Search Strategy

A structured literature review was conducted to identify relevant studies on recent advances in regenerative endodontics. Electronic databases, including PubMed, Scopus, and Consensus, were searched using keywords such as “regenerative endodontics”, “tissue engineering in endodontics”, “dental pulp stem cells”, “scaffolds in endodontics”, “biomaterials for pulp regeneration”, and “clinical protocols in regenerative endodontics”. The search was conducted without time restrictions to capture both foundational studies and the latest advancements, although preference was given to articles published in the last 15 years to ensure relevance to current clinical practices and research.

Inclusion and Exclusion Criteria

To ensure relevance and quality in the selection of studies, specific inclusion and exclusion criteria were applied, as summarized in Table 1.

**Table 1:** Inclusion and exclusion criteria

Criteria	Inclusion	Exclusion
Scope of study	Studies focusing on regenerative endodontic techniques, including stem cell applications, scaffold materials, and disinfection protocols	Studies unrelated to endodontics or without relevance to pulp-dentin regeneration
Type of research	Experimental and clinical studies, including in vitro, in vivo, and clinical trials; comprehensive narrative and systematic reviews	Non-experimental articles, such as editorials or opinions, unless offering significant insights into future research needs
Quality of publication	Articles published in peer-reviewed journals	Non-peer-reviewed sources, such as informal publications or preprints, unless relevant to emerging topics
Relevance to objectives	Studies providing insights into the efficacy, challenges, and future directions of regenerative endodontics	Studies focusing exclusively on conventional endodontic therapies without any regenerative approach
Publication date	Priority to studies published in the last 15 years to capture current techniques, with consideration of older foundational studies	Older studies only if they lack relevance to contemporary advances or were superseded by recent findings
Outcome focus	Reports on clinical outcomes such as apical closure, root maturation, pulp vitality restoration, and dentin thickness improvement	Studies lacking outcome data relevant to regeneration, such as those solely focused on microbiology or unrelated pathologies

Data Extraction and Analysis

Key areas for data extraction and analysis were identified to systematically examine regenerative endodontic techniques. Table 2 summarizes these areas, covering stem cell applications, scaffold materials, signaling molecules, clinical protocols, and outcome measurements.

**Table 2:** Key areas for data extraction and analysis

Key area	Description	Focus of extraction
Stem cell types and applications	Information on various stem cell types, their origins, and applications in regenerating the pulp-dentin complex	Type, origin, and application in pulp-dentin complex regeneration
Scaffold materials and design	Details on scaffold materials and design innovations to support cellular growth, differentiation, and organization	Material type (e.g., collagen, hydrogels), structural properties, design innovations
Signaling molecules	Types of signaling molecules used, including growth factors and cytokines, and their roles in guiding tissue formation	Molecule types, delivery mechanisms, roles in tissue formation
Clinical protocols and disinfection methods	Protocols for canal disinfection and challenges in maintaining an environment for stem cell survival and differentiation	Disinfection agents, methods, stem cell viability support
Outcome measurements	Clinical outcomes such as apical closure, root maturation, pulp vitality restoration, and dentin thickness	Key clinical outcomes related to regenerative success and structural improvements

Quality Assessment and Risk of Bias

To ensure reliability, each selected study was assessed for quality and potential biases. Experimental studies were evaluated for sample size, methodology consistency, and reproducibility. Clinical studies and trials were reviewed for risk of bias, particularly concerning patient selection, blinding, and outcome reporting. A modified version of the Cochrane Risk of Bias tool was used to categorize studies as having low, moderate, or high risk of bias. Only studies with low to moderate risk of bias were included in the analysis to ensure high-quality evidence.

Data Synthesis

Data synthesis was organized into thematic categories based on the tissue engineering triad-stem cells, scaffolds, and signaling molecules, as well as clinical protocols. Qualitative analysis was used to interpret findings across studies, highlighting patterns, challenges, and variations in clinical outcomes. Key studies and their contributions to advancing regenerative endodontics were summarized in tables to enhance clarity and accessibility. The results of this synthesis provide a comprehensive overview of current advances, challenges, and potential future directions in regenerative endodontics, laying the foundation for practical recommendations and research needs in the field.

Review

Stem Cell Applications in Regenerative Endodontics

Stem cells are central to regenerative endodontics due to their unique ability to differentiate into specialized cell types necessary for forming the pulp-dentin complex. The primary stem cell types used in regenerative endodontics include DPSCs, SCAPs, PDLSCs, and DFPCs [1]. Each stem cell type offers distinct advantages; DPSCs, for example, derived directly from the pulp, have a high potential for odontogenic differentiation, making them ideal for dentin regeneration [6]. SCAPs, located in the apical papilla of immature teeth, are capable of differentiating into root dentin cells, which is crucial for promoting root development in immature teeth with necrotic pulp [10].

In addition to DPSCs and SCAPs, PDLSCs and DFPCs are being studied for their applications in pulp-dentin regeneration. PDLSCs show potential for periodontal ligament and pulp repair, offering flexibility in their use across various regenerative applications [6]. Stem cells are harvested either directly from dental tissues or through biobanking, where extracted cells are preserved for future use, allowing for patient-specific treatments [8].

Experimental studies in animal models have shown that DPSCs and SCAPs transplanted into root canals can lead to the formation of a pulp-dentin complex with essential structural characteristics [5]. However, despite promising results, clinical translation remains challenging due to variability in stem cell viability and the risk of immune rejection in allogenic applications [3].

Gene-modified stem cells, where specific genes are activated to improve cell survival, are another area of ongoing research to enhance regenerative outcomes [7]. Despite progress, standardized protocols for stem cell use in endodontics are still lacking, emphasizing the need for further studies to establish guidelines for selecting optimal cell types and preparation methods [14].

The characteristics and applications of various stem cell types used in regenerative endodontics are detailed in Table 3.

**Table 3:** Characteristics and applications of stem cell types in regenerative endodontics

Stem cell type	Source	Application in endodontics	Advantages	Challenges
Dental pulp stem cells (DPSCs)	Dental pulp tissue	Dentin regeneration and pulp tissue formation	High odontogenic potential	Limited availability in mature teeth
Stem cells from apical papilla (SCAPs)	Apical papilla	Root dentin formation, root lengthening in immature teeth	Vitality in immature teeth	Limited to immature teeth only
Periodontal ligament stem cells (PDLSCs)	Periodontal ligament	Periodontal regeneration and pulp repair	Supports periodontal ligament repair	Less studied in endodontic applications
Dental follicle progenitor cells (DFPCs)	Dental follicle	Pulp-dentin complex regeneration	High proliferative potential	Limited availability and research focus

Scaffold Materials in Regenerative Endodontics

Scaffolds are crucial in regenerative endodontics as they provide a supportive matrix that promotes cellular growth, differentiation, and organization. An ideal scaffold must be biocompatible, biodegradable, and mimic the natural extracellular matrix of pulp tissue. Common scaffold types include natural polymers like collagen and chitosan, synthetic polymers like polycaprolactone, polylactic acid, and hydrogels [4]. Collagen-based scaffolds are widely used due to their biocompatibility and structural similarity to pulp tissue, which facilitates cell adhesion and migration [11].

Hydrogels have gained popularity in regenerative endodontics due to their high water content, which supports nutrient diffusion and waste removal, creating an optimal environment for cellular activity [13]. Bioengineered hydrogels can encapsulate growth factors, enabling controlled release to guide cell differentiation and tissue development [2]. Additionally, studies on polycaprolactone and other synthetic polymers have demonstrated that these materials support stem cell viability while providing customizable degradation rates [8].

Challenges in scaffold development include optimizing properties such as pore size, mechanical strength, and degradation rate to align with tissue formation [10]. Scaffolds must also be bioactive to promote the migration and adhesion of stem cells and support differentiation into odontoblast-like cells [9].

The properties and applications of different scaffold materials used in regenerative endodontics are presented in Table 4.

**Table 4:** Properties and applications of scaffold materials in regenerative endodontics

Scaffold material	Type	Properties	Advantages	Challenges
Collagen	Natural polymer	Biocompatible, supports cell adhesion and migration	Mimics natural extracellular matrix, widely used in endodontics	Limited structural strength
Chitosan	Natural polymer	Antimicrobial properties, biocompatible	Supports cell growth and differentiation	Variable degradation rate
Polycaprolactone	Synthetic polymer	Biodegradable, tunable mechanical properties	Supports stem cell viability	Slow degradation in the body
Hydrogels	Synthetic/natural	High water content, can encapsulate growth factors	Promotes nutrient diffusion, customizable	Structural weakness, challenging handling

Signaling Molecules in Regenerative Endodontics

Signaling molecules are integral to regenerative endodontics as they direct stem cell differentiation and tissue growth. Important signaling molecules in this field include bone morphogenetic proteins (BMPs), vascular endothelial growth factor (VEGF), and transforming growth factor-beta (TGF-β). BMPs play a critical role in inducing odontogenic differentiation and promoting dentin formation [1]. VEGF, on the other hand, enhances vascularization, which is essential for establishing a healthy pulp tissue environment [6].

These molecules are often delivered within scaffolds or through controlled-release systems to ensure sustained bioavailability in the root canal [3]. Advanced scaffolds that release these molecules in response to environmental cues are under investigation to provide a localized stimulation of cellular activity. However, achieving optimal timing, dosing, and control remains challenging, as imbalances can lead to inflammation or abnormal tissue growth [2].

Gene therapy, where genes encoding specific growth factors are directly introduced into cells, offers a promising approach to achieving consistent signaling molecule delivery [5]. This approach remains experimental, yet it may provide sustained and controlled release of molecules essential for regeneration [8].

Signaling molecules essential for guiding cell differentiation and tissue formation in regenerative endodontics are outlined in Table 5.

**Table 5:** Functions and delivery methods of key signaling molecules in regenerative endodontics

Signaling molecule	Function	Delivery method	Advantages	Challenges
Bone morphogenetic proteins (BMPs)	Promotes odontogenic differentiation	Scaffold-based release	Supports dentin formation	Requires precise dosing
Vascular endothelial growth factor (VEGF)	Stimulates blood vessel formation	Controlled-release hydrogels	Enhances tissue vascularization	Potential for over-stimulation
Transforming growth factor-beta (TGF-β)	Cell proliferation and differentiation	Gene therapy, microspheres	Broad role in cell growth and immune modulation	Inflammatory response in high doses
Fibroblast growth factor (FGF)	Cell growth and proliferation	Injectable systems	Promotes cellular proliferation and differentiation	Short half-life, challenging delivery

Clinical Protocols and Disinfection Techniques

A critical aspect of regenerative endodontics is the development of clinical protocols that ensure both effective disinfection and a supportive environment for tissue regeneration. Disinfection is typically achieved using agents such as sodium hypochlorite, EDTA, and chlorhexidine, which help eliminate pathogens while preparing the canal walls for cell adhesion [12]. Sodium hypochlorite, while highly effective, can be cytotoxic, posing risks to stem cell survival if used at high concentrations [1].

Alternative disinfection methods, such as light-activated disinfection and laser-assisted irrigation, offer enhanced cleaning capabilities while reducing the need for potentially harmful chemicals [13]. Additionally, the triple antibiotic paste (metronidazole, ciprofloxacin, and minocycline) is widely used due to its broad-spectrum antimicrobial effects, although it can also negatively impact stem cell viability [7].

Optimizing these protocols to balance disinfection and biocompatibility remains a focus of ongoing research. Newer methods, such as low-concentration sodium hypochlorite followed by EDTA rinses, are designed to minimize cytotoxicity, promoting stem cell survival and maximizing regenerative potential [6].

Various disinfection methods and protocols used in regenerative endodontics are summarized in Table 6.

**Table 6:** Disinfection methods and protocols in regenerative endodontics

Signaling molecule	Function	Delivery method	Advantages	Challenges
Bone morphogenetic proteins (BMPs)	Promotes odontogenic differentiation	Scaffold-based release	Supports dentin formation	Requires precise dosing
Vascular endothelial growth factor (VEGF)	Stimulates blood vessel formation	Controlled-release hydrogels	Enhances tissue vascularization	Potential for over-stimulation
Transforming growth factor-beta (TGF-β)	Cell proliferation and differentiation	Gene therapy, microspheres	Broad role in cell growth and immune modulation	Inflammatory response in high doses
Fibroblast growth factor (FGF)	Cell growth and proliferation	Injectable systems	Promotes cellular proliferation and differentiation	Short half-life, challenging delivery

Future Directions and Emerging Techniques

Recent advancements in regenerative endodontics show promising directions in gene therapy, advanced scaffold designs, and cell-free regenerative approaches. Gene therapy, in particular, holds the potential for more precise control over cellular behavior by enabling targeted delivery of growth factors to the pulp-dentin complex. Gene-modified cells can express signaling molecules essential for directing differentiation and tissue formation, though its application in endodontics is still largely experimental [15].

Innovations in 3D bioprinting technology allow for the development of customized scaffolds that better mimic the natural architecture of pulp tissue. This technique can enable more effective cellular alignment and nutrient flow within the root canal environment, potentially improving the outcomes of pulp-dentin regeneration [16]. Studies have indicated that 3D bioprinted scaffolds may offer improved structural integration and support the gradual release of bioactive molecules, thus enhancing the overall regenerative process [17].

Another promising area of research is the exploration of cell-free regenerative approaches, which rely on bioactive scaffolds and growth factor delivery systems rather than directly transplanting stem cells. Clinical trials on cell-free therapies have demonstrated comparable efficacy to cell-based methods, with reduced risks of immune rejection and ethical concerns associated with stem cell use [18]. These approaches may streamline the regenerative process, making it more accessible and cost-effective for broader clinical applications [19].

Furthermore, studies are increasingly focusing on creating standardized protocols for regenerative endodontics that can be universally applied in clinical settings. These protocols would address the variability in regenerative outcomes by setting guidelines for disinfection methods, scaffold selection, and the use of signaling molecules [20]. Establishing these protocols will be essential to ensuring consistency in clinical outcomes and advancing regenerative endodontics from experimental to mainstream practice.

The various future directions and emerging techniques in regenerative endodontics are summarized in Table 7.

**Table 7:** Future directions and emerging techniques in regenerative endodontics

Technique	Description	Potential advantages	Challenges
Gene therapy	Utilizes genetic modification to introduce growth factor genes into cells to enhance regeneration	Precise control over cellular behavior, targeted tissue repair	Experimental; risk of off-target effects, immune responses
3D bioprinting	Uses bioprinting technology to create customized, biomimetic scaffolds that replicate pulp tissue	Enhanced structural integration, supports cell alignment and nutrient diffusion	Equipment complexity; high cost, accessibility limitations
Cell-free regeneration	Relies on bioactive scaffolds and growth factors without direct stem cell transplantation	Reduced immune rejection risk, simplified protocol	Potentially less effective than cell-based methods in some cases
Standardized protocols	Establishes universal clinical guidelines for regenerative techniques	Consistent outcomes, easier mainstream clinical adoption	Achieving consensus; variable patient responses

Advances in Biomaterials for Regenerative Endodontics

Advances in biomaterial science have introduced new materials that support pulp-dentin regeneration. Recently, bioactive materials such as calcium silicate-based materials, nanofibrous scaffolds, and injectable hydrogels have been developed to provide not only structural support but also bioactive properties that encourage cell adhesion and differentiation [21]. Calcium silicate-based materials like MTA and biodentine have shown promising results in regenerating the pulp-dentin complex by forming a tight seal that encourages hard tissue formation [22].

Nanofibrous scaffolds, made from materials such as polylactic-co-glycolic acid (PLGA), support stem cell growth due to their high surface area and similarity to the natural extracellular matrix [23]. Studies suggest that nanofibrous scaffolds may improve pulp tissue regeneration outcomes by enhancing cell differentiation and biomineralization [24]. Injectable hydrogels, incorporating bioactive peptides or nanoparticles, have also gained attention due to their ease of application and ability to encapsulate growth factors for controlled release [25].

Immunomodulation in Regenerative Endodontics

Immunomodulation, or the strategic management of immune responses, is becoming an important factor in regenerative endodontics. The immune response plays a dual role, aiding in pulp repair but potentially disrupting regenerative processes if uncontrolled [26]. Macrophages, for example, can adopt different activation states, from pro-inflammatory (M1) to anti-inflammatory (M2), influencing the healing outcomes of regenerative procedures [27].

Research has shown that modulating macrophage polarization to the M2 state can enhance tissue repair and reduce inflammation in the root canal environment [28]. Studies on the use of anti-inflammatory agents or immune-modulatory biomaterials, such as corticosteroids or cytokine inhibitors, suggest the potential for improving regenerative outcomes by creating a more favorable environment for stem cell survival and differentiation [29].

Clinical Outcomes and Long-Term Success Rates of Regenerative Endodontic Procedures

Evaluating the long-term success and clinical outcomes of REPs is critical to understanding their viability as routine treatments. Studies have shown that REPs can achieve successful apical closure, root elongation, and dentin wall thickening in immature teeth [30]. However, some studies indicate variability in outcomes depending on factors such as patient age, type of stem cells used, and procedural differences [31].

Research following patients treated with REPs over several years has shown mixed results, with success rates generally favorable in younger patients and cases with immature roots [6]. However, cases in mature teeth or with complex root anatomy often face challenges in achieving comparable regenerative outcomes [32].

## Conclusions

Regenerative endodontics marks a significant shift in dental care, moving beyond conventional preservation techniques to biologically restore the pulp-dentin complex. Advances in stem cell research, scaffold development, and signaling molecules offer promising avenues for restoring both structure and function in non-vital teeth. Despite current challenges such as variability in outcomes and the need for standardized protocols, ongoing research in biomaterials, gene therapy, and bioprinting holds great potential to overcome these limitations and enhance the reliability and effectiveness of regenerative endodontics for long-term dental restoration. While regenerative endodontics is still evolving, it stands to transform dental practice by providing biologically restorative solutions that prioritize long-term tooth vitality and function.
